# Effectiveness of a Peer-Led Web-Based Intervention to Improve General Self-Efficacy in Using Dating Apps Among Young Adults: Randomized Clustered Trial

**DOI:** 10.2196/16378

**Published:** 2020-10-30

**Authors:** William CW Wong, Wai Han Sun, Shu Ming Cheryl Chia, Joseph D Tucker, William PH Mak, Lin Song, Kitty Wai Ying Choi, Stephanie Tsz Hei Lau, Eric Yuk Fai Wan

**Affiliations:** 1 Department of General Practice University of Hong Kong-Shenzhen Hospital Shenzhen China; 2 Department of Family Medicine and Primary Care University of Hong Kong Hong Kong China; 3 Sticky Rice Love Ltd. Hong Kong China; 4 University of North Carolina at Chapel Hill - Project China Guangzhou China; 5 London School of Hygiene and Tropical Medicine London United Kingdom; 6 Vocational Training Council Hong Kong China; 7 Department of Communication Faculty of Social Sciences University of Macau Macau Macao; 8 Department of Pharmacology and Pharmacy University of Hong Kong Hong Kong China

**Keywords:** internet, sexual health, self-efficacy, young adult, risk assessment

## Abstract

**Background:**

Online dating apps are popular platforms for seeking romance and sexual relationships among young adults. As mobile apps can easily gain access to a pool of strangers (“new friends”) at any time and place, it leads to heightened sexual health risks and privacy concerns.

**Objective:**

This study aimed to evaluate the effectiveness of a peer-led web-based intervention for online dating apps to prepare Chinese college students so that they have better self-efficacy when using dating apps.

**Methods:**

An open clustered randomized controlled trial was conducted among students from three colleges (The University of Hong Kong, Hang Seng University of Hong Kong, and Yijin Programme of Vocational Training College) in Hong Kong. Students aged 17 to 27 years who attended common core curriculum or general education were randomized into intervention and control groups. The intervention material, developed with high peer engagement, included four short videos, an interactive scenario game, and a risk assessment tool. An existing website promoting physical activities and healthy living was used as a control. Using the information, motivation, and behavioral skills (IMB) approach to design the evaluation, questionnaires covering participants’ sociodemographics and dating app characteristics, as well as the general self-efficacy scale (GSE) as the primary outcome and the risk propensity scale (RPS) as the secondary outcome were administered before, immediately after, and at 1 month after the intervention. Intention-to-treat analysis was adopted, and between-group differences were assessed using the Mann-Whitney *U* test. A post-hoc multiple linear regression model was used to examine the correlates of the GSE and RPS.

**Results:**

A total of 578 eligible participants (290 in the intervention group and 288 in the control group) participated in the study with 36 lost to follow-up. There were more female participants (318/542, 58.7%) than male participants in the sample, reflecting the distribution of college students. Over half of the participants (286/542, 52.8%) reported the following reasons for using dating apps: being curious (170/498, 34.1%), trying to make new friends (158/498, 31.7%), and finding friends with similar interests (121/498, 24.3%). Overall, the participants in the intervention group reported favorable experiences when compared with the finding in the control group. There was significant improvement in the GSE score and reduction in the RPS score (*P*<.001) in the intervention group. University of Hong Kong students were more susceptible to risk reduction after the intervention when compared with students from the other two institutions.

**Conclusions:**

The online intervention was effective in improving general self-efficacy and reducing risk tendency among young students. Future work is needed to determine if this approach is cost-effective and such behavioral change is sustainable.

**Trial Registration:**

ClinicalTrials.gov NCT03685643; https://clinicaltrials.gov/ct2/show/NCT03685643.

**International Registered Report Identifier (IRRID):**

RR2-10.1186/s13063-018-3167-5

## Introduction

### Background of Online Dating Apps

The use of online dating apps has become popular for seeking friendship and romantic and sexual relationships owing to the advancement of mobile technology and increased internet accessibility through smartphones. Many dating apps are free to download and embed a geolocation function that allows users to find nearby like-minded individuals. Through virtual communication platforms, users can easily meet friends and gain access to a wide pool of potential sexual partners at any place and time.

In the United States, there was an increase in the use of online or mobile dating apps by 4% in only 2 years (from 11% in 2013 to 15% in 2015) [1]. Young adults (aged 18-24 years) and older adults (aged 50-60 years) contributed most to the increase in dating app usage [1]. A more recent survey conducted in 2017 showed that 30% of adults aged 18 to 29 years were using dating apps [2]. Sumter and Vandenbosch found that 17% of young adults had been using popular mobile dating apps, such as Tinder, Grindr, and Coffee meets Bagel in the past 2 years [3]. Online dating apps are also gaining popularity among Chinese youth [4]. It was reported that as many as two-fifth of millennials used online dating apps in Hong Kong [5]. Young adults (including university students) have been identified as the largest population group engaged in online dating.

Prior research found an increased level of risky sexual behavior among individuals who sought multiple partners through online domains [6]. According to Sawyer et al, young adults accounted for an estimated 50% of the 20 million new sexually transmitted infection (STI) cases per year, and less than half of them used condoms [7]. A local study found that dating app users were more likely to have multiple sexual partners and unprotected sex, resulting in an increased risk of STI [6]. Impulsivity has been consistently associated with risky sexual behavior, which contributes to the increasing rates of STI among young adults [8]. The characteristics of impulsive individuals usually involve living in the moment and less likelihood of delaying gratification. Hence, they are more likely to engage in activities that are seen as “fun” and “exciting,” which are the characteristics of meeting someone online. Dating apps with built-in geolocation and instant messaging functions facilitate inner impulsivity among young adults to have social and even sexual interactions. In the case of dating apps, the lack of prescreening and instilling essential knowledge, attitudes, and behaviors among users could cause emerging concerns beyond public health. In our previous study, we found that dating app users could experience other nonhealth-related concerns, such as financial scams and privacy intrusion [9]. As sex education is not compulsory in Hong Kong schools but is provided on an ad hoc basis or as an extracurricular activity [10], the lack of training and awareness regarding dating app use could further expose these young people to the aforementioned risks.

### Peer-Led Approach and Crowdsourcing in Intervention Development

Nowadays, many young adults gain sexual knowledge from their peers or the internet. Therefore, peer-led education, which is defined as “teaching or sharing of health information, values, and behaviors between individuals with shared characteristics such as social status, experience, and cultural backgrounds” [11], could be an effective strategy for disseminating sex education. It is an integrative approach that involves delivery of an intervention by peers of similar age in settings, such as community centers and youth clubs, using pedagogical or “diffusional” methods. Our systematic review and meta-analysis on peer-led sex education in developed countries demonstrated that peer-led education could effectively change sexual health knowledge and attitudes among young people [12].

In this study, a crowdsourcing method was adopted to form the backbone of this peer-led approach. Crowdsourcing is defined as having a group to solve a problem [13]. It is a tool that uses a bottom-up approach to facilitate and engage the community. Crowdsourcing allows a diverse group of individuals, including laypeople and experts, to contribute to public health interventions [14]. In addition to innovation, crowdsourcing discourages cognitive fixation, a process where people become focused upon others’ ideas in a group setting, but forms a basis to allow development of promotional videos and images in a cooperative manner.

In collaboration with the research team and a nongovernmental organization (NGO) that specializes in online sex education (*StickyRiceLove*), a crowdsourcing contest called “‘Hi, Stranger!’ Dating Apps Education Design Contest” was conducted from December 2017 to April 2018 among students from the University of Hong Kong (HKU) enrolled in a common core course (Sexuality and Culture), as well as the members of a local NGO network called *Sexuality Education Alliance.* They were first asked to generate and submit ideas in the form of texts, images, videos, and websites. They were later evaluated based on the content, interactivity, creativity, and credibility by a team of experts made up of public health physicians and sociologists using a peer-vetted creative production approach [15]. The following five key risk domains were identified: sexual harassment, privacy, monetary issues, legal issues, and mental well-being. The synthesized results were used to develop the content and modes of delivery of the intervention in a design workshop by the peer volunteers from *StickyRiceLove*. Full details of the intervention development are published elsewhere [9].

### Study Aim and Hypothesis

The aim of this study was to assess the effectiveness of a peer-led web-based intervention among young adults enrolled in three tertiary educational institutions in Hong Kong. The specific objectives were to evaluate the effectiveness and the participants’ acceptability of the peer-led online intervention based on the changes in participants’ emotions, awareness, attitudes, and behavioral skills in using dating apps. In addition, this study explored participants’ sociodemographic factors and dating app characteristics as moderators of the primary outcome of the intervention (ie, general self-efficacy scale [GSE] score) and the secondary outcome (risk propensity scale [RPS] score). It was hypothesized that there would be a statistical difference between the intervention and control groups, in which the participants in the intervention group would have higher self-efficacy and a lower tendency to take risks 1 month after the intervention. In addition, differences in the education environment and mode of study among the tertiary institutions would affect the GSE and RPS scores.

## Methods

### Study Design

An open, clustered, randomized controlled trial (RCT) involving a peer-led safer sex intervention and control (1:1 allocation) was conducted among college students between September 2018 and May 2019. Allocation concealment was achieved through the sealed envelope method [16], and class clusters were randomly assigned to the two groups by an independent researcher. The study protocol and its design were approved by the Institutional Review Board of Hong Kong West Cluster and HKU (UW 18-369).

### Sampling Methods

The inclusion criteria were as follows: (1) age 17 to 27 years and (2) first- or second-year college students enrolled into different tutorial groups or classes in the common core curriculum or general education courses in the three main tertiary educational institutions based in Hong Kong, namely the HKU, Hang Seng University of Hong Kong (HSUHK), and Yijin Programme of Vocational Training College. Participants were excluded if they were color blind, had no access to a computer/internet literacy, and could not read/write Chinese.

The HKU is the oldest tertiary educational institution in Hong Kong and is regarded as one of the most prestigious universities in Asia, with five out of six applicants admitted being in the top 10% achievement ranking in the Hong Kong Examinations and Assessment Authority public examination. HSUHK is a nonprofit self-financed private university that offers undergraduate and taught postgraduate degrees, with five schools in the areas of business, communication, decision sciences, humanities and social science, and translation. The Yijin Programme is an alternative pathway for college education of generic skills including language, interpersonal skills, and communication offered to individuals who have completed high school or adult learners aged 21 years or above. Its qualification is accepted by government agencies as meeting academic entry requirements for civil service posts.

Based on the primary outcome of self-efficacy, a previous cluster RCT on school-delivered rights-based sexuality education for adolescents revealed a standard deviation of 0.56 and effect size of 0.20 [17]. Considering a minimal cluster size of 10 students, level of significance of .05, power of 0.8, and intracluster correlation coefficient of 0.007, at least 338 students were required for this study, with a 30% assumed attrition rate.

### Intervention

The final intervention involved four (2-4 min) short videos, an interactive scenario game, and a risk assessment tool (Multimedia Appendix 1, Multimedia Appendix 2, Multimedia Appendix 3, and Multimedia Appendix 4). The videos aimed to address the risks and benefits of using dating apps and encouraged the viewers to reflect on their perception of dating apps [9]. The first video illustrated similarities between meeting people on dating apps and in real life, providing examples of being misled by profile characteristics and monetary scams. The second video mainly targeted privacy concerns. The third demonstrated risky sexual behaviors associated with dating apps, and the fourth explained the legal issues and risks of sexual assault, including precautionary steps and available resources.

**Figure 1:**
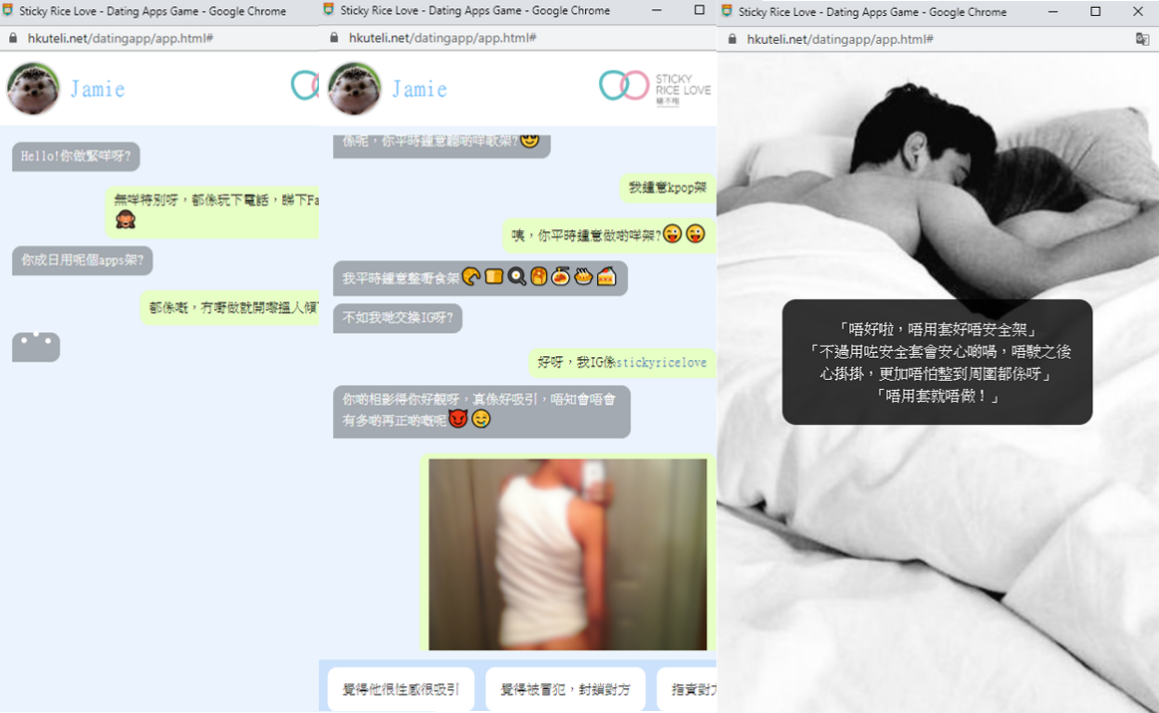
Screenshots of the online game.

The scenario game depicted in Figure 1 is a first-person simulation game where the participant is presented with multiple choices when faced with real-life scenarios [9]. The game has been designed with various algorithms that result in both positive and adverse outcomes. A brief rationale for how player choices resulted in the outcomes would be indicated. Lastly, a risk assessment evaluation consisting of 14 questions would appear, and it would give the participant a score to infer the risk level for adverse events when using dating apps.

#### Intervention Procedure

According to the protocol, participants were informed about the voluntary nature of the assessment and that their refusal to participate would not affect their grades [18]. The participating students were asked not to share or discuss the interventions at the time of enrollment. A quasianonymous approach was used (ie, no login when accessing the intervention on the computer/mobile phone). Technical or logistical measures (eg, cookies, email confirmation, and phone calls) were not used to detect or prevent access.

After obtaining written consent, participants completed a preintervention online questionnaire, a postintervention questionnaire immediately after the intervention, and a follow-up questionnaire that encompassed questions relating to risk assessment 1 month later. The questionnaires were constructed and administered using a password-secured survey tool called SurveyGizmo to be accessed by a QR code using participants’ mobile devices at their institutions. A reminder was sent to the students who did not complete the follow-up questionnaire after 1 week, and the questionnaire was closed 2 weeks after delivery. Upon completion, participants were given a HK $50 (US $1=HK $7.8) coffee coupon as gratitude for participation.

In contrast, an existing Hong Kong government website promoting physical activity and healthy living was used in the control group. The “health exercise for all” campaign website has a similar modality as that in the intervention group (ie, videos and interactive games) to illustrate the different forms of exercise for different age groups and settings [19]. The game allows players to input personal information, including weight, gender, sports preference, and time spent on an individual sport with a personalized training diary and exercise tips.

#### Evaluation

The effectiveness of the intervention was guided by the information (facts, heuristics, and implicit theories), motivation (personal and social), and behavioral skills (self-efficacy and objective skills) (IMB) model [20]. The GSE includes 10 items for assessing one’s ability to cope with a range of difficulties in daily life, correlated to one’s self-belief, emotion, optimism, and work satisfaction [21]. The RPS rates general risk-taking tendencies with nine items on a 9-point Likert scale. A higher score indicates higher risk-taking tendencies [22]. Patient Health Questionnaire-2 (PHQ-2) is a two-item scale to assess the frequency of depressed mood and anhedonia over the previous 2 weeks, with preliminary screening for depressive symptoms [23]. In our study, the self-reported GSE was considered an appropriate primary outcome measure, as the GSE not only measures one’s beliefs about their capabilities to control life events, such as finding a relationship or sexual partner online and achieving desirable outcomes, but is also related to social anxiety such that a negative GSE score indicates vulnerability to higher levels of stress when facing a difficult situation [24].

The preintervention questionnaire consisted of sociodemographic factors and dating app characteristics, such as age, gender, sexual orientation, relationship status, housing type, social media use, reasons to engage in dating apps, addiction risk, and unhappy encounters associated with dating apps (Multimedia Appendix 5). The postintervention questionnaire encompassed questions on acceptability of the intervention (ie, expressed interest, appropriateness of content, sufficient examples and explanation provided, cultivation of the participants’ skills, continual use, and likelihood of recommendation to friends) (Multimedia Appendix 6). The follow-up questionnaire included questions matching those in the preintervention questionnaire, with additional questions regarding visitation frequency and actual recommendation to friends (Multimedia Appendix 7).

### Statistical Methods

The demographics were described, and differences in baseline were assessed using chi-square tests for categorical variables (eg, age, gender, and sexual orientation) and *t* tests for scaled outcomes. In order to conduct a chi-square test for the evaluation of the intervention’s acceptability among the participants, five scaled options were categorized into two groups as follows: “strongly agree, agree, and neutral” and “strongly disagree and disagree.” Students who did not complete the questionnaire were included for the analysis as per intention to treat (ITT), with “last observation carried forward” values adopted. Owing to the skewness distribution of the outcomes, the differences of intervention testing for mainly the GSE, RPS, and PHQ-2 at follow-up were assessed using the Mann-Whitney *U* test. Univariate linear regression and multiple linear regression models were used to measure the association among the different confounding factors (eg, gender, relationship status, and reasons for using dating apps) against the primary and secondary outcomes. All data were entered, cleaned, and analyzed using SPSS (version 25; IBM Corp), and statistical significance was set at a *P* value <.05.

## Results

### Demographics and Dating App Statistics

**Figure 2:**
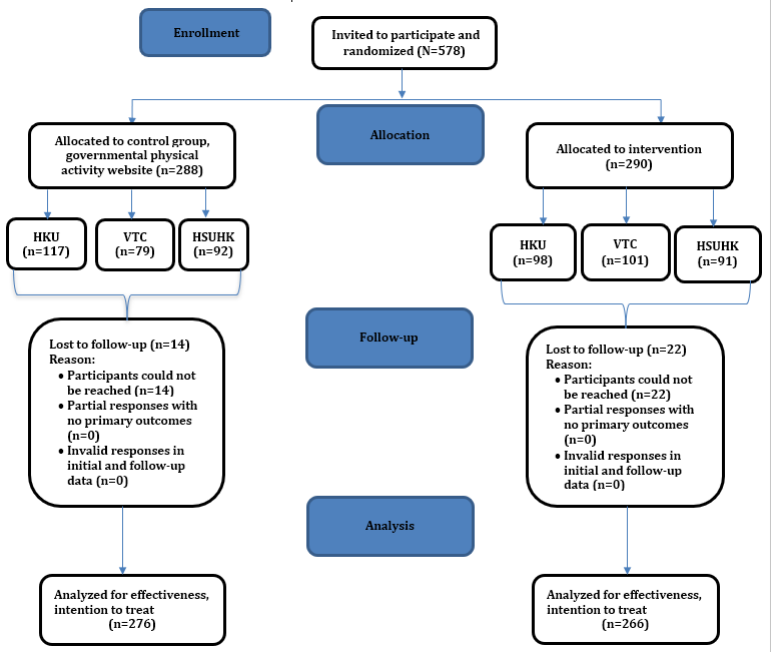
CONSORT flow diagram. HKU: University of Hong Kong; HSUHK: Hang Seng University of Hong Kong; VTC: Vocational Training College.

A total of 578 participants who completed the preintervention questionnaire were randomized into the intervention (n=290) and control (n=288) groups. Since common core curriculum and general education classes were compulsory courses, the response rate was generally very high (215/240, 89.5% for HKU; 183/200, 91.5% for HSUHK; and 180/208 for Yijin Programme, 86.5%). A total of 36 participants were lost to follow-up, giving rise to a dropout rate of 6.2% (Figure 2).

Participants in the intervention and control groups had a similar age profile (ie, mean 19.83 years, SD 1.9 years and mean 19.88 years, SD 1.8 years, respectively) (Table 1). There were more females (318/542, 58.7%) than males (224/542, 41.3%), reflecting the gender ratio in tertiary education (53.3% females and 46.7% males pursuing undergraduate studies in 2017/2018, University Grants Committee). Most of the participants were heterosexual (490/542, 90.4%). Two-thirds of the recruited participants were single (353/542, 65.1%), and the remaining (189/542, 34.9%) were in some form of relationship. In addition, about one-third stayed at private accommodation (208/542, 38.4%) or public housing (186/542, 34.3%).

Table 2 presents the gathered responses pertaining to the participants’ characteristics regarding dating apps. Nearly half of the recruited participants (286/578, 49.5%) were found to have visited dating websites. The participants suggested that internet (258/606, 42.6%) and friends (266/606, 43.9%) were the most popular means to gain information about dating apps according to a multiple response setting where the participants could select more than one choice per question. In addition, social media was highlighted by the responses as the most popular social networking platform across both the intervention and control groups (96/218, 44.0% and 101/225, 44.9%, respectively).

Being curious (170/498, 34.1%), trying to make new friends (158/498, 31.7%), and finding friends with similar interests (121/498, 24.3%) were popular selections made by the participants regarding what attracted them to engage in dating apps. Most of the dating app users responded that they did not face any discontent (207/286, 72.4%), except for a minority that faced privacy intrusion (34/286, 11.9%) and sexual assailment (23/286, 8.0%) before. More than half of the responses (169/285, 59.3%) highlighted that using dating apps to establish friendship required a longer time than anticipated. The participants perceived that dating apps had affected their work or school life (106/285, 37.2%), sleep (125/285, 43.9%), and relationship with their friends/family (75/285, 26.3%). At baseline, no relevant differences were found among the outcomes across the intervention and control groups (*P*=.09 to *P*=.85).

**Table 1:** Baseline sociodemographic characteristics of the participating college students.

Variable		Intervention group (N=290), n (%) or mean (SD)	Control group (N=288), n (%) or mean (SD)
**Gender**			
	Female	139 (47.9%)	179 (62.2%)
	Male	137 (47.2%)	87 (30.2%)
	Not specified	14 (4.8%)	22 (7.6%)
Age (years)		19.83 (1.9)	19.88 (1.8)
**Type of housing**			
	Private apartment	107 (36.9%)	101 (35.1%)
	Public housing estates	104 (35.9%)	82 (28.5%)
	Subsided housing	34 (11.7%)	39 (13.5%)
	Student hostel	19 (6.6%)	29 (10.1%)
	Village	7 (2.4%)	15 (5.2%)
	Short interim	5 (1.7%)	0 (0%)
	Not specified	14 (4.8%)	22 (7.6%)
**Relationship status**			
	Single	183 (63.1%)	170 (59.0%)
	Others (dating, married, or cohabitating)	93 (32.1%)	96 (33.3%)
	Not specified	14 (4.8%)	22 (7.6%)
**Sexual orientation**			
	Heterosexual	250 (86.8%)	240 (83.3%)
	Others (gay, lesbian, or bisexual	24 (8.3%)	26 (9.0%)
	Not specified	14 (4.9%)	22 (7.6%)

**Table 2:** Characteristics of the responses from participants about their dating app use.

Variable			Intervention group (N=290), n (%) or mean (SD)	Control group (N=288), n (%) or mean (SD)	*P* value^a^
**Ever used a dating website**					.51
	Yes		139 (47.9%)	147 (51.0%)	
	No		151 (52.1%)	141 (49.0%)	
**Source of information about dating apps**					
	Internet		118 (34.8%)	140 (52.4%)	<.001
	Friends		147 (43.3%)	119 (44.6%)	.82
	Teachers or school staff		52 (15.3%)	4 (1.5%)	<.001
	Magazine or fashion materials		3 (0.9%)	2 (0.7%)	.75
	Family/parents		19 (5.6%)	2 (0.7%)	<.001
**Social networking platforms**					
	Social media platform (eg, Facebook and Instagram)		96 (44.0%)	101 (44.7%)	.49
	Dating apps		42 (19.3%)	77 (62.1%)	<.001
	Instant chatline (eg, WhatsApp and WeChat)		78 (35.8%)	43 (34.7%)	<.001
	Others (eg, school and online game)		2 (0.9%)	4 (3.2%)	.03
**Reasons for using dating apps**					
	Making new friends		73 (29.9%)	85 (33.5%)	.38
	Out of curiosity		83 (34.0%)	87 (35.6%)	.77
	Finding friends with similar interest		61 (25.0%)	60 (24.5%)	.54
	Sexual relationship		11 (4.5%)	9 (3.7%)	.59
	Long-term relationship		16 (6.6%)	13 (5.3%)	.39
**Encounter of unhappiness while using dating apps**					.77
	Yes		38 (27.3%)	41 (27.9%)	
	No		101 (72.6%)	106 (72.1%)	
**Types of problem encountered with dating apps**					
	Financially scammed		7 (18.4%)	5 (12.5%)	.65
	Cyberbully/blackmail		5 (12.3%)	4 (10.0%)	.74
	Privacy issues		15 (39.5%)	19 (47.5%)	.86
	Sexual assault		11 (28.9%)	12 (30.0%)	.73
**Dating apps and their addiction implications**					
	**Time spent on dating app is longer than anticipated**				.20
		Disagree	61 (44.2%)	55 (37.4%)	
		Agree	77 (55.8%)	92 (62.6%)	
	**Affect work or school life**				.30
		Disagree	83 (60.1%)	96 (65.3%)	
		Agree	55 (39.9%)	51 (34.7%)	
	**Affect sleep**				.83
		Disagree	77 (55.8%)	83 (56.5%)	
		Agree	61 (44.2%)	64 (43.5%)	
	**Affect relationship with friends/family**				.67
		Disagree	104 (25.2%)	107 (72.8%)	
		Agree	35 (74.8%)	40 (27.2%)	
**Outcomes**					
	GSE^b^		23.95 (5.25)	22.65 (4.85)	.27
	RPS^c^		5.96 (0.95)	5.97 (0.90)	.85
	PHQ-2^d^		3.86 (1.33)	4.10 (1.38)	.09

^a^Chi-square test is used to compare the statistical difference in categorical variables between groups. Mann-Whitney *U* test is also used.

^b^GSE: general self-efficacy scale.

^c^RPS: risk propensity scale.

^d^PHQ-2: Patient Health Questionnaire.

### Intervention Evaluation Outcomes

As presented in Table 3, the responses received indicated that the proportion of participants who became interested in the dating program was significantly higher in the intervention group (162/410, 39.5%) than in the control group (88/410, 21.5%; *P=*.002). In addition, the proportion of participants who responded that the intervention provided sufficient explanations and examples was significantly higher in the intervention group (231/410, 56.3%) than in the control group (129/410, 31.5%; *P*<.001). Moreover, the proportion of participants who responded that they would recommend the approach to their friends was significantly higher in the intervention group (211/410, 51.5%) than in the control group (125/410, 30.5%; *P=*.04). Furthermore, one-quarter of participants from the intervention group (114/497, 23.0%) and one-tenth of participants from the control group (47/497, 9.5%) had introduced the intervention to their friends (*P*<.001).

After adjustment for the baseline GSE score, a significant difference was found between the intervention and control groups at the 1-month follow-up (*P*<.001). A similar result trend is elicited especially in HSUHK, where a significant difference was observed (*P*<.001). In addition, a small but significant (*P=*.04) decrease in the GSE score by −0.03 for every 1 unit increase could be observed in the control group (Table 4).

Using the stepwise univariate regression analysis, dating apps and their addiction implications were found to have a positive association for affecting the RPS score (*P*<.001) (Table 5). On the other hand, other factors having positive associations for affecting the GSE score were relationship status (*P=*.02), reasons to use dating apps, in particular, seeking sexual relationships (*P*=.002) and long term relationships (*P=*.014), information about dating apps disseminated by teachers/school staff (*P=*.02), and differences in gender (*P=*.04). These significant factors were further analyzed using a backward multiple regression analysis, and it was found that there was a significant difference in the treatment group for the GSE score (*P*<.001) and RPS score (*P*<.001) (Table 6). In addition, HKU was found to be a significant confounding factor (*P*=.006) negatively associated with the RPS score.

**Table 3:** Feedback responses at postintervention.

Variable		Intervention group (N=276), n (%)	Control group (N=266), n (%)	*P* value^a^
**This intervention caused me to be interested in the dating program.**				
	Disagree	86 (34.6%)	74 (45.7%)	.002
	Agree	162 (65.3%)	88 (54.3%)	
**The content of the intervention is appropriate.**				
	Disagree	17 (6.9%)	18 (11.1%)	.14
	Agree	231 (93.1%)	144 (88.9%)	
**This intervention provided sufficient explanations and examples.**				
	Disagree	17 (6.9%)	33 (20.4%)	<.001
	Agree	231 (93.1%)	129 (79.6%)	
**This intervention has cultivated my ability and skills.**				
	Disagree	44 (17.7%)	33 (20.4%)	.52
	Agree	204 (82.3%)	129 (79.6%)	
**I will continue to use the intervention.**				
	Disagree	39 (9.5%)	37 (9.0%)	.07
	Agree	209 (50.1%)	125 (30.5%)	
**I will recommend this intervention to my friends.**				
	Disagree	37 (15.7%)	37 (22.8%)	.04
	Agree	211 (84.3%)	125 (77.2%)	
**Are there any beneficial effects with respect to the program?**				
	Yes	209 (90.1%)	188 (81.7%)	.16
	No	23 (9.9%)	42 (18.3%)	
**Are there any improvements to be made to the program?**				
	Yes	123 (60.6%)	141 (61.5%)	.72
	No	80 (39.4%)	88 (38.4%)	
**Have you visited the HKU^b^ intervention website after the study?**				
	Yes	153 (56.0%)	130 (57.0%)	.09
	No	120 (43.9%)	98 (43.0%)	
**Have you recommended any friends after the intervention?**				
	Yes	114 (42.7%)	47 (20.4%)	<.001
	No	153 (57.3%)	183 (79.6%)	

^a^Chi-square test is used to compare the statistical difference between groups.

^b^HKU: University of Hong Kong.

**Table 4:** Effectiveness of the intervention in participating college students at the 1-month follow-up.

Scale	Intervention (N=290), mean (SD)			Control (N=288), mean (SD)			Control vs intervention *P* value				Univariate regression analysis *P* value (95% CI)
	HKU^a^ (n=98)	HSUHK^b^ (n=91)	VTC^c^ (n=101)	HKU (n=117)	HSUHK (n=92)	VTC (n=79)	HKU	HSUHK	VTC	Total	
GSE^d^	24.55 (5.57)	24.68 (4.85)	23.59 (5.88)	23.01 (4.71)	22.32 (5.14)	23.37 (5.87)	.12^e^	<.001^e^	.53^e^	<.001^f^	.04 (−0.03 to −0.01)
RPS^g^	5.57 (0.99)	5.67 (1.01)	4.72 (1.55)	5.73 (1.09)	5.65 (1.13)	5.31 (1.23)	.10^e^	.97^e^	.32^e^	.12^f^	.06 (0.02 to 0.09)
PHQ-2^h^	4.24 (1.41)	3.78 (1.34)	4.55 (1.52)	4.09 (1.31)	3.77 (1.15)	4.30 (1.51)	.47^e^	.80^e^	.26^e^	.19^f^	.23 (−0.05 to 0.01)

^a^HKU: University of Hong Kong.

^b^HSUHK: Hang Seng University of Hong Kong.

^c^VTC: Vocational Training College.

^d^GSE: general self-efficacy scale.

^e^Mann-Whitney *U* test is used to compare the statistical difference between groups within institutions.

^f^Mann-Whitney *U* test is used to compare the statistical difference between groups overall.

^g^RPS: risk propensity scale.

^h^PHQ-2: Patient Health Questionnaire-2.

**Table 5:** Factors affecting the general self-efficacy scale and risk propensity scale scores using a univariate regression model.

Factor			General self-efficacy scale score				Risk propensity scale score			
			Coefficient β (95% CI)		*P* value^a^		Coefficient β (95% CI)		*P* value^a^	
**Sociodemographic factors**										
	Age			0.00 (−0.23 to 0.23)		.98		−0.02 (−0.08 to 0.03)		.34
	**Gender** (ref^b^: male)									
		Female		−0.93 (−1.79 to −0.06)		.04		0.11 (−0.09 to 0.32)		.28
	**Housing type** (ref: public housing)									
		Subsidized housing		0.13 (−1.11 to 1.36)		.84		0.19 (−0.11 to 0.48)		.22
		Private housing		−0.51 (−1.38 to 0.36)		.25		−0.20 (−0.41 to 0.01)		.06
		Short interim		1.40 (−3.88 to 6.66)		.60		−0.10 (−1.49 to 1.29)		.87
		Student hostel		−0.35 (−1.88 to 1.18)		.66		0.21 (−0.11 to 0.56)		.19
		Village		2.08 (−0.06 to 4.21)		.06		−0.25 (−0.75 to 0.26)		.34
	**Sexual orientation** (ref: heterosexual)									
		Gay/lesbian/bisexual		−0.09 (−1.56 to 1.38)		.90		0.02 (−0.33 to 0.37)		.15
	**Relationship status** (ref: single)									
		Dating/marriage/cohabitating		0.10 (0.12 to 1.88)		.03		−0.15 (−0.36 to 0.06)		.37
**Dating characteristics**										
	**Ever used dating apps** (ref: yes)									
		No		−0.69 (−1.53 to 0.14)		.10		0.13 (−0.06 to 0.33)		.18
	**Types of platforms to know friends** (ref: dating apps)									
		Social media platforms and instant chatline		−1.37 (−2.91 to 0.18)		.08		−0.01 (−0.40 to 0.38)		.96
	**Reasons for using dating apps** (ref: curiosity)									
		New friends		−0.62 (−1.87 to 0.63)		.33		−0.11 (−0.41 to 0.19)		.47
		Similar interest		−0.32 (−1.53 to 0.89)		.60		−0.23 (−0.07 to 0.53)		.14
		Sexual relationship		3.58 (1.36 to 5.80)		.002		0.23 (−0.36 to 0.81)		.45
		Long-term relationship		2.38 (0.46 to 4.27)		.01		−0.13 (−0.62 to 0.37)		.61
	**Types of media** (ref: friends)									
		Internet		−0.01 (−0.90 to 0.87)		.98		0.05 (−0.16 to 0.26)		.63
		Teachers or school staff members		−1.72 (−3.17 to −0.27)		.02		−0.06 (−0.41 to 0.28)		.72
		Magazine or fashion materials		0.11 (−4.28 to 4.50)		.96		0.66 (−0.48 to 1.79)		.26
		Family/parents/government initiation		0.18 (−2.04 to 2.39)		.88		−0.01 (−0.63 to 0.43)		.71
	**Dating apps and their online implications**									
		Affect sleep		−0.12 (−0.62 to 0.39)		.65		0.26 (0.12 to 0.40)		<.001
		Spent a longer time than usual on a dating app		−0.25 (−0.76 to 0.26)		.84		0.35 (0.16 to 0.54)		<.001
		Affect work or school		−0.31 (−0.87 to 0.25)		.28		0.31 (0.13 to 0.49)		<.001
		Affect relationship with friends and family		−0.61 (−1.21 to 0.00)		.05		0.37 (0.15 to 0.56)		<.001

^a^*P* value is analyzed using univariate analysis.

^b^ref: reference.

**Table 6:** Factors affecting the general self-efficacy scale and risk propensity scale scores using a multiple linear regression model.

		General self-efficacy scale score		Risk propensity scale score	
		Coefficient β (95% CI)	*P* value^a^	Coefficient β (95% CI)	*P* value^a^
Intervention group		−2.79 (−4.65 to −0.92)	.004	0.453 (0.099 to 0.81)	.01
**Gender** (ref^b^: male)					
	Female	−1.56 (−3.42 to 0.30)	.98	N/A^c^	N/A
**School** (ref: VTC^d^)					
	HSUHK^e^	−0.03 (−4.86 to 4.81)	.99	−0.37 (−0.79 to 0.06)	.09
	HKU^f^	0.35 (−2.93 to 3.63)	.83	−0.62 (−1.06 to −0.18)	.006
**Baseline outcomes**					
	General self-efficacy scale	−0.51 (−0.68 to −0.34)	<.001	N/A	N/A
	Risk propensity scale	N/A	N/A	−0.39 (−0.55 to −0.22)	<.001

^a^*P* value is analyzed using multiple linear regression analysis.

^b^ref: reference.

^c^N/A: not applicable.

^d^VTC: Vocational Training College.

^e^HSUHK: Hang Seng University of Hong Kong.

^f^HKU: University of Hong Kong.

## Discussion

### Principal Findings

To the best of our knowledge, there has been limited research focusing on the awareness of online dating app risks among young adults. Most sex-related apps and dating apps contain no information about sexual health promotion [25]. This finding is consistent with the finding of another local study that the use of dating websites was common among Hong Kong college students, mostly for curiosity and seeking new friends [26]. The participants from the intervention group disclosed having a greater interest toward the peer-led intervention compared with participants from the control group. They felt that clear and sufficient examples were given as illustrations compared with participants from the control group. For outcome evaluation, the findings supported that the intervention could elicit significant changes in participants’ GSE skills (*P*=.04), as well as significant changes in risk-taking tendencies (*P*=.06).

In our study, participants’ GSE scores increased after the intervention, although the absolute difference was small. The small increase in the GSE score was important as an indication that the web-based intervention changed their understanding of perceived control over the usage of dating apps in a short duration of time. A positive GSE finding may also indicate that participants were less anxious about being “judged” by potential partners. They may feel less anxious owing to their ability to hold positive appraisals [24]. Young adults with higher GSE scores would feel more efficacious and less anxious when using dating apps. The promising result of our study regarding enhanced general self-efficacy toward online dating app use is consistent with the findings of a systematic review stipulating that high levels of peer participation in these types of interventions could greatly improve sex knowledge, attitude, general self-efficacy, and social norms [12]. The positive outcome exhibited could be related to the introduction of gamification in nongaming situations that made the intervention unique and appealing to the targeted youth. The use of real-life scenarios and humor in games and videos may have attracted and retained the participants’ attention [27]. As a result, this motivated and improved users’ experience and engagement [28]. This was in contrast to a slight deterioration in the GSE score by −0.02 for every 1 unit increase in the control group, suggesting that the impertinent content relating to physical activity was unable to provide knowledge and skills in relation to safer dating app use.

A significant negative change was found in the risk propensity tendency among the participants overall, especially among HKU participants (*P*=.006) (Table 6), suggesting that the web-based intervention may influence young adults who already have strong interest in sexuality and sexual health or that more academically capable students may be more amicable to the intervention. Coupled with the strong emphasis of the potential adverse sexual health outcomes and the precautionary steps from the web-based intervention [25], the intervention may help students to be more cautious toward online dating apps. Such results were not observed among students from other colleges for whom the motivation for unsafe behavior could be attributed more strongly to benefits than to risk perception. On the other hand, it is well known that risk perception itself is insufficient to explain the risky behavior [29].

According to analysis of the online search threads, it was found that young adults may have a higher tendency to engage with the digital world to search for sexual health concerns; topics including sexual pleasure, puberty, menstruation, and transmission; and mechanisms of infection [27]. Therefore, with the successful implementation of our intervention, we hope that people of all ages, not just young adults, could benefit from this new innovative way of training on how to use dating apps safely.

### Strengths and Limitations

The major strengths of the study include its randomized design, low attrition rate, and implementation of validated outcome measures to assess the effectiveness of our peer-led web-based intervention. The game stimulation allows participants to create a personal profile that can drive tailoring functions and personalized messages. With the incorporation of risks and benefits in this peer-led web-based intervention, it could serve as a practical approach to shape and instill online dating app users with right values.

In contrast, the participants recruited in the selected universities may not be representative of the whole youth population. Further, peer-led education is effective in establishing safer sex norms and attitudes than adult-led interventions, but less so in imparting factual knowledge [30]. Although self-reported measures are common and practical methods to obtain information in behavioral health studies, self-reporting bias may be introduced, especially under-reporting the sensitive nature of sexual behavior [31]. Multiple entries were still possible despite the prohibition of duplicate responses from identical IP addresses. Lastly, the limited timeframe and exposure of the intervention may make it unable to capture any long-term changes in the intensity, frequency, or context of participants’ emotions. Therefore, a longer intervention period is required for significant and sustainable changes in outcomes at the risk of high attrition rates. In theory, contamination could occur as participants from the control and intervention groups attended the same class, which could result in undermining of the effect magnitude of the intervention.

### Implications and Recommendations

With the increasing popularity of mobile technology and the internet, this multifaceted intervention could serve as a starting point for novel interventions to increase safety awareness when seeking sexual partners online. Continuing to document dating app users’ experiences through testimonials or videos demonstrated on the intervention website may increase general self-efficacy and reduce the risk tendency toward dating app use. The results obtained from the study could be insightful for public health practitioners and app companies to improve the safety profile of dating apps. Furthermore, novel delivery methods, such as social media and community-based centers, could be considered to reach out to an extensive youth population beyond the college setting. Health professionals could also share and update any new research findings pertaining to the detrimental health impacts associated with casual sex to deter users from risky sexual behavior through dating apps and to create a long-lasting unfavorable outlook about it. Lastly, trial-based health economic evaluation was not conducted in this study, and a previous study showed that a peer-led approach could be potentially more expensive than an adult-led initiative (i.e. €28.2 [US $33.8] per target student in the peer-led group vs €11.6 [US $13.9] in the teacher-led arm) [32]. Therefore, it is important to further investigate the cost-effectiveness, barriers, opportunities, and supporting measures to sustain the intervention in the long term by placing more emphasis on feedback from all stakeholders (eg, students, teachers, and members of the school) to allow the development of programs that are culturally and socially appropriate.

### Conclusion

This study demonstrated that a peer-led intervention could improve short-term self-efficacy and reduce risk propensity in using dating apps among young Chinese adults. It is important to evaluate the motivations for using dating apps and attempt to understand the underlying mechanism between using dating apps and associated risks. Future work is needed to determine how to maintain behavioral change over a longer duration, how to reach underserved populations using different means, and how to disseminate such an intervention on a large scale.

## Appendix

Multimedia Appendix 1 Video illustrating similarities between meeting people on dating apps and in real life. Examples of being misled by profile characteristics and monetary scams.

Multimedia Appendix 2 Video depicting privacy concerns related to dating apps.

Multimedia Appendix 3 Video depicting risky sexual behavior.

Multimedia Appendix 4 Video depicting legal issues and risks of sexual assault, including precautionary steps and available resources.

Multimedia Appendix 5 Preintervention questionnaire.

Multimedia Appendix 6 Postintervention questionnaire.

Multimedia Appendix 7 Follow-up intervention questionnaire.

## Abbreviations

GSE: general self-efficacy scale
HKU: University of Hong Kong
HSUHK: Hang Seng University of Hong Kong
NGO: nongovernmental organization
PHQ-2: Patient Health Questionnaire-2
RCT: randomized controlled trial
RPS: risk propensity score
STI: sexually transmitted infection
